# Outdoor environments and human pathogens in air

**DOI:** 10.1186/1476-069X-8-S1-S15

**Published:** 2009-12-21

**Authors:** Ka man Lai, Jean Emberlin, Ian Colbeck

**Affiliations:** 1 Department of Civil, Environmental and Geomatic Engineering, University College London, London WC1E 6BT, UK; 2 National Pollen and Aerobiology Research Unit, University of Worcester, Henwick Grove, Worcester WR2 6AJ, UK; 3 Department of Biological Sciences, University of Essex, Wivenhoe Park, Colchester CO4 3SQ, UK

## Abstract

Are pathogens in outdoor air a health issue at present or will they become a problem in the future? A working group called AirPath - *Outdoor Environments and Human Pathogens in Air *was set up in 2007 at University College London, UK with the aim of opening new discussion and creating a research network to investigate the science and impacts of outdoor pathogens. Our objective in this paper is to review and discuss the following areas: What is the source of human pathogens in outdoor air? What current, developing and future techniques do we need? Can we identify at-risk groups in relation to their activities and environments? How do we prepare for the anticipated challenges of environmental change and new and emerging diseases? And how can we control for and prevent pathogens in outdoor environments? We think that this work can benefit the wider research community and policy makers by providing a concise overview of various research aspects and considerations which may be important to their work.

## Introduction

Low moisture and nutrient levels, combined with high levels of ultraviolet (UV) radiation mean that the atmosphere is inhospitable to microbial life [1]. The huge volume of air outdoors compared to air indoors also helps to dilute the concentration of microbes and reduce the level of exposure. Nevertheless, we need to ask: are pathogens in outdoor air a health issue at present or will they become problematic in the future? The multidisciplinary working group, AirPath was organised to review and discuss the problem of pathogens. Four two-day meetings over a period of 18 months from July 2007 have generated contributions from more than 30 participants, over 20 oral presentations, and various round-table discussions, which were recorded on DVD. In order to summarise the wealth of knowledge contributed by the multidisciplinary group, the panel (the authors of the present paper) has arranged the discussion into five themes: 1. What is the source of human pathogens in outdoor air? 2. What current, developing and future techniques do we need? 3. Can we identify at-risk groups in relation to their activities and environments? 4. How do we prepare for the anticipated challenges of environmental change and new and emerging diseases? and 5. How can we control for and prevent pathogens in outdoor environments?

Participants were selected on the basis of their involvement, for example, as president or executive committee members of related professional and academic bodies such as the British Aerobiology Federation, the Aerosol Society and the International Association of Aerobiology; their work in related UK organisations, such as the Health Protection Agency (HPA), hospitals and Defence Science and Technology Laboratory (DSTL); or their research in relevant fields such as epidemiology, meteorology, geoinformatics, and natural resource management. Researchers outside the UK were invited to give an international dimension and network of collaboration to the UK participants. Rather than focus on a narrow topic, the aim of the AirPath working group is to explore the complex and multidisciplinary facets of research connected with the outdoor environment and human pathogens in air, analyse their potential implications, and investigate applications of this research for the well being of society.

## Research methods, review and discussion

At the first two meetings, participants were given a topic and asked to prepare a one-page literature review to support their presentations; each topic was assigned to two participants from different disciplines in order to ensure cross-disciplinarity of the discussion. These presentations now form the core of our review, and all the discussions from all the meetings were recorded. We have conducted an extended literature search and review to ensure that the content is representative, comprehensive and connected to the five main areas arranged below.

### What is the source of human pathogens in outdoor air?

**Figure 1:**
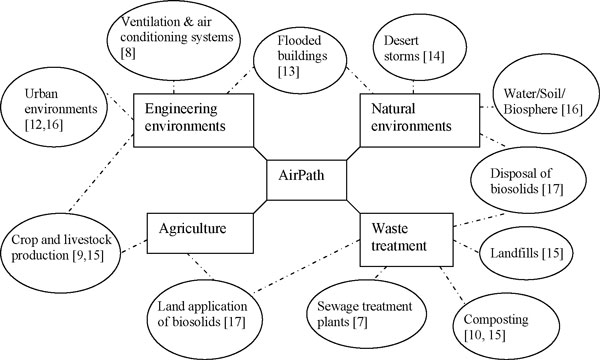
**Environmental sources of pathogens in outdoor air**.

Participants discussed four main areas that are known to contribute pathogens to the outdoor air and have proved to be linked with human health - 1. Natural environments 2. Engineering environments 3. Agriculture and 4. Waste treatment (Figure 1). As shown in Figure 1, air pathogens from environmental sources are diverse in terms of type of source and aerosolisation factors, and it is moreover possible that some pathogens are under-reported because a number of them cause similar respiratory symptoms, e.g. coughing and sneezing [2,3]. Because there are numerous types of pathogens released to the outdoors and given the publishing constraints, the list of pathogens from different sources can be found in the cited references in Figure 1. In addition, although many environmental pathogens are restricted to limited geographic areas, it may be naïve to assume that pathogens previously restricted to specific locations will not shift with impending global climate change; or we may find that our current knowledge is based on inadequate data and we have yet to discover the actual distribution of a number of these pathogens [2,4]. The pathogens cited in the references may not yet be relevant to the UK, but could pose a future threat due, for example, to climate change. Moreover, various pathogens can potentially be released from waste treatment facilities; how will the mounting levels of composted green waste, food waste and other traditional landfill materials, as well as increasing bio-solid applications to land, impact on the pathogens in air?

### What current, developing and future techniques do we need?

What types and how many pathogens are we exposed to? Is our environment changing and impacting on our health? To answer these questions, we require various techniques to support our research, such as sampling, detection and identification, monitoring, transport models, and laboratory experimentation (Table 1). Kuske reviewed the current and emerging technologies for the study of bacteria in outdoor air in 2006 [5]. We searched the literature from 2006 onwards to extend Kuske's review and paid close attention to the study of viruses. Some studies show that climatological factors can influence viral disease transmission, e.g. respiratory syncytial virus [6]. The environmental sampling of human viruses is therefore an area that we regard as important in order to prepare for new and emerging diseases in our time.

**Table 1:** Current, developing and future techniques with a highlight on the study of viruses

Techniques	Description
Sampling	Sampling of bioaerosols (bacteria and fungi) is widely reviewed [5]. Bioaerosols can be collected on various media depending on the type of microbial detection, and can be collected according to their size to estimate their deposition on the respiratory system. All of these sampling techniques have pros and cons regarding the issues of size separation, sampling volume and time, biological recovery, and particle removal efficiency as well as the choice of subsequent analytical and detection methods. The sampling and quantification of viruses is less widely studied. One recent study has developed methods for airborne influenza and avian influenza virus, which is currently one of the biggest concerns of public health [11].

Detection and Identification	Detection and identification of pathogens has changed since the development of different molecular methods and innovative approaches other than culture methods [5]. The existing detection methods can be divided into two levels: generic and specific. Generic detection gives information about whether the particles are biological materials, microbes or living cells, e.g. bioluminescent measurement of ATP using continuous flow luminometer and mass-spectrometry. Specific methods such as micro-arrary and immuno-assays can tell us what kind of microbes are detected and identified. Other new techniques have been proposed for bio-detection, for instance, by characterising the size and shape of bioaerosols, pollens and fungal spores under microscope [18] and analysing fluorescence spectrum of bacteria [5].

Monitoring	It is widely recognised that background biological and chemical materials and their continuous environmental fluctuation will significantly influence monitoring. Air movement, sunlight/UV radiation, humidity, rainfall, and inversions are some of the environmental factors that need to be considered during monitoring. Another consideration is where and when to sample with regard to spatial and temporal relevance [19]. For example, the release of pathogens can cause a significant downwind hazard which requires a wide area and long period of sampling [7].

Transport/Transmission models	Epidemiology studies can link disease cases together and develop a disease transport and transmission model [8]. However, it will not always explain the mechanism. Moreover, it requires a significant number of cases in order to develop a model. The use of computational fluid dynamic (CFD) models and tracer gas simulation has demonstrated that the Severe Acute Respiratory Syndrome (SARS) virus can travel and disperse outdoors through air, and became a source of pathogens to other indoor environments [12]. A similar technique has been used in the modelling of aerosols and chemical pollutants in streets, waste treatment facilities, and other pathogen sources outdoors [7].

Biological Experimentation	Because pathogens travel in air, it is inevitable that the biological activity will be influenced by the environment. Data can be collected from field studies to determine the impact of environments on the fate and behaviour of pathogens. However, since the pathogens and environment vary and fluctuate frequently, it is not easy to build this scientific link using field data alone. Some studies have investigated the viability and environmental limits of airborne viruses and bacteria using a rotating drum and controlled climate environmental chambers [20].

Table 1 highlights the diverse skills and knowledge outside the traditional microbiology and aerobiology fields, which can be adopted to understand pathogens in the outdoor air. Kuske's review [5] contributes largely with regard to sampling and detection and identification in Table 1. Our review has added, for example, the spatial and temporal monitoring issues, epidemiology and computational fluid dynamic models, and the application of laboratory experimental facilities. Moreover, a variety of examples were given on the study of viruses in the air.

### Can we identify at-risk groups in relation to their activities and environments?

It is important to better understand the risk factors associated with the outdoor air transmission of infections, but the best approach is not always straightforward. Epidemiology is the study of the link between exposure, outcome and confounders, but the measurement of exposure to outdoor pathogens in the air is difficult, as it is problematic to conduct controlled laboratory experiments, for example, to expose people to pathogens within the laboratory. In addition, the technology to obtain accurate organism counts may not yet be available. Moreover, it will be a challenge to evaluate the possible environmental influences such as climatic conditions and proximity to a source, when we assess exposure levels, especially retrospectively [6-8]. Furthermore, it is not always clear how the outcomes should be measured [9,10]. Many respiratory infections do not have a definitive causal organism [3]. Infections may be asymptomatic and most infections have numerous subtypes [3,8]. Age, gender, social class, health, exposure to pollution, and a variety of other factors are the potential confounders that must be addressed in future epidemiology studies.

From the AirPath point of view, to carry out an exposure assessment - that is, to estimate and measure the amount of infectious pathogens entering our bodies through inhalation - is already a complex and multidisciplinary science without the added component of using exposure data and disease outcomes to predict risk factors. We think that AirPath has contributed towards forming a technical network, as shown in the output in Table 1, as well as a medical network to further clarify a comprehensive and systematic exposure assessment.

### How do we prepare for the anticipated challenges of environmental change and new and emerging diseases?

Studies from both field and laboratory settings indicate that environments and environmental factors can significantly impact on the fate and behaviour of bioaerosols and health risk. It is generally recognised that the environment is constantly changing, either physically, climatically, socially, or a combination of all three. Not only is the environment ever changing, but the types of diseases are also changing. The presence of new and emerging diseases is one of the most urgent threats to humanity across the globe [3,11,12]. Climate change is a high priority issue that everyone is facing; and it may be that environmental pathogens will respond to climate change as well. Climate change not only affects the pathogens in air, but also their source, source strength and aerosolisation mechanisms (e.g. through extreme weather conditions) [13,14]; these are fairly unexplored at present.

New and emerging diseases are a major health concern because we do not know much about them. We do not yet know how best to prevent and control them and, most importantly, we often do not know how to treat them, but it is likely that genetics and the state of the immune system of the global population plays a key role in disease prevalence [2]. With advances in medical treatment, greater numbers of disease-susceptible groups, such as immuno-compromised individuals are expected to survive longer in the overall population. This trend may change our understanding of pathogens because many unexpected microbes and infection pathways can appear within this transforming population.

### How can we control for and prevent pathogens in outdoor environments?

Once pathogens are in the air, one of the few things we can do to minimise the health risk is to source control (reduce/eliminate the source and its strength and the aerosolisation factors, as well as potential dispersal interception near the source) [15]. Modelling the movement of bioaerosols in order to advise people to prepare or even evacuate is another option for reducing the health risk [10]. Although the physical properties and transport of aerosols can be predicted in most environments, it is not always known how biological properties change in the aerosol dynamics pathway and during outdoor transport (e.g. aggregation, scavenging and deposition). Source control is not always possible, for instance, if the sources of pathogens are unknown. The longer it takes to identify the source, the higher the risk it poses to people.

Because of the differences in the type of sources, the nature of the pathogens and their geographical prevalence, various research and policy approaches have been taken by different regulatory bodies. Using Legionellosis as an example, guidelines and regulations are available from various government agencies, cooling tower manufacturers and industrial trade organisations to control and prevent the growth and dispersal of these particular pathogens. Although guidelines and regulations are widely known and implemented, outbreaks of Legionnaire's disease are not uncommon. Some studies report that the pathogens can travel up to 12 km from the suspected source and still cause infections [8], thus indicating a more complicated relationship between source and disease transmission than was previously understood.

There is a significant knowledge gap in understanding and assessing the risk of outdoor pathogens in air. In order to complete the transmission pathway and set up regulation, control and prevention measures, we need to better understand and identify pathogens at their source, identify the aerosolisation mechanisms and dispersal plume, have adequate qualitative and quantitative techniques to detect the pathogens in different media, and understand the deposition process and required dose for infection. As in Skyes et al.'s analysis, a risk assessment framework was used to assess the potential health risks from bioaerosols from composting [10]. Current knowledge of pathogen risk assessment in air is far from complete; without knowing the infectious dose and actual risk to society, there is little motivation on the part of governments or manufacturers to research methods of control.

## Conclusion

In our complex, changing world, some environmental health problems are not straightforward to identify until a very serious health impact occurs. When health problems do emerge, we may only have a very short period of time and opportunity in which to react, identify and restore the damaged natural environment. As a result, we strongly assert the necessity to understand the environment and its relationship with pathogens in air before health hazards associated with the relevant pathogens can appear. In the present paper we have briefly reviewed and presented our views on various issues. Our next step is to encourage and support focused multidisciplinary research in order to fill the missing knowledge gaps and translate research into practice and policy.

## Note

The peer review of this article can be found in Additional file 1.

## Competing interests

The authors declare that they have no competing interests.

## Authors' contributions

Dr. Ka-man, Lai is the PI of the AirPath working group. She led the meetings and preparation of this paper. Prof. Jean Emberlin and Prof. Ian Colbeck are the Co-I of this working group. They advised on the topics for discussion in the meetings and in this paper as well as helped to make connection to the appropriate experts and organisations to participate in the group.

## Supplementary Material

Additonal file 1 Peer review. Click here for file
