# Supplementary material for: Outdoor environments and human pathogens in air
Source: Environ Health. 2009 Dec 21;8(Suppl 1):S15. doi: 10.1186/1476-069X-8-S1-S15 (PMC2796493; doi:10.1186/1476-069X-8-S1-S15)
Supplement: Additonal file 1 — Peer review. [file 1476-069X-8-S1-S15-S1.doc]

| Article ref no. | A1471-2105X SZ-S15 |
| --- | --- |
| Title | **Outdoor environments and human pathogens in air** |
| Author(s) | Ka man Lai, Jean Emberlin and Ian Colbeck for AirPath |

**Ref#1**

**General comments:**

This paper summarizes the work of the Airpath network. The network has been active and successful in bringing together scientists from a range of disciplines to discuss the important topic of pathogens in outdoor air. The authors of this paper have attempted to present the broad range of subject matter discussed by the network and to focus attention on some specific aspects of particular importance. In the context of a short paper this is a challenging task. The authors have structured their review of the network’s work according to a set of key research questions. I have commented according to these subheadings;

***What are the human pathogens in outdoor air?***

- Fig 1 presents a useful overview of the environmental sources of pathogens in outdoor air
- The rest of this section does little to identify pathogens of concern related to those environmental sources identified. In fact only two pathogens are mentioned.

***What do we need - current, developing and future techniques?***

- Table 1 presents a review of issues surrounding techniques for viruses (although the caption does not mention viruses)
- The text describing this table could be improved – is it possible to pull out key conclusions drawn from this review of techniques?

***Risk factors - humans, environments and activities?***

- This is a very interesting section. However it contains two questions regarding challenges for exposure assessment and controlled experiments that are not explained or developed and as such their importance or meaning is not clear.

***Prepare for the new challenge: environmental change and new and emerging diseases***

- No comments. The issues relating to environmental change are important.

***Control and prevention in outdoor environments***

- Please check sentence 1 – it seems to contain a contradiction.

**Could the authors please review the comments made and make changes accordingly? Also please re-check the manuscript for English.**

**Ref#2**

**General comments:**

This is a particularly poor paper, lacking clarity of method, reasoning and rigour. Reviews, opinion pieces and/or the outcomes of expert panel discussions are often really useful publications. Usually however, such pieces are clear in their methods and approach and the reader knows how supporting literature was identified or how the expert discussion was captured, how the material has been analysed and therefore what the opinions in the paper actually represent. Without this clarity or rigour, the paper takes on the feel of one person’s outpouring of opinion.

For example, we are told the review focuses on ‘the recent examples’; how are these defined? What does recent mean? Was the literature systematically searched for and analysed? How was it assessed? What criteria were used to selected the cited examples? I like the idea of reporting discussion, but how was the discussion structured? When did it take place? Was it prompted? Were the topics decided *a priori*? Was it recorded? How did the members decide which bits of the discussion to include in this paper?

My feeling is that the authors have something useful and interesting to say, but to make a persuasive case to which the wider scientific audience will pay attention and value requires a higher standard of presentation and rigour.

## Responses to reviewers’ comments

**Ref#1**

***What are the human pathogens in outdoor air?***

- Fig 1 presents a useful overview of the environmental sources of pathogens in outdoor air

**No change in Fig 1**

- The rest of this section does little to identify pathogens of concern related to those environmental sources identified. In fact only two pathogens are mentioned.

**The text was changed according to this comment**

**p.4 What is the source of human pathogens in outdoor air? “Because there are numerous types of pathogens released to the outdoors and given the publishing constraints, the list of pathogens from different sources can be found in the cited references in Figure 1.”**

***What do we need - current, developing and future techniques?***

- Table 1 presents a review of issues surrounding techniques for viruses (although the caption does not mention viruses)

**The caption of table 1 was changed “Table 1**. **Current, developing and future techniques with a highlight on the study of viruses “**

- The text describing this table could be improved – is it possible to pull out key conclusions drawn from this review of techniques?

**A paragraph was added in p.5 “Table 1 highlights the diverse skills and knowledge outside the traditional microbiology and aerobiology fields,…..”**

***Risk factors - humans, environments and activities?***

- This is a very interesting section. However it contains two questions regarding challenges for exposure assessment and controlled experiments that are not explained or developed and as such their importance or meaning is not clear.

**For controlled environments, an example was added to explain what is difficult to be done in the laboratory “controlled laboratory experiments, for example to expose people to pathogens within the laboratory,..”**

**A paragraph was added to explain exposure assessment p.6, “From the AirPath point of view, to carry out an exposure assessment - ….. and systematic exposure assessment. “**

***Prepare for the new challenge: environmental change and new and emerging diseases***

- No comments. The issues relating to environmental change are important.

***Control and prevention in outdoor environments***

- Please check sentence 1 – it seems to contain a contradiction.

**We changed the title to “How can we control for and prevent pathogens in outdoor environments?”**

**Could the authors please review the comments made and make changes accordingly?**

**Also please re-check the manuscript for English. Yes, changed and checked.**

**Ref#2**

**General comments:**

This is a particularly poor paper, lacking clarity of method, reasoning and rigour. Reviews, opinion pieces and/or the outcomes of expert panel discussions are often really useful publications. Usually however, such pieces are clear in their methods and approach and the reader knows how supporting literature was identified or how the expert discussion was captured, how the material has been analysed and therefore what the opinions in the paper actually represent. Without this clarity or rigour, the paper takes on the feel of one person’s outpouring of opinion.

For example, we are told the review focuses on ‘the recent examples’; how are these defined? What does recent mean? Was the literature systematically searched for and analysed? How was it assessed? What criteria were used to selected the cited examples? I like the idea of reporting discussion, but how was the discussion structured? When did it take place? Was it prompted? Were the topics decided *a priori*? Was it recorded? How did the members decide which bits of the discussion to include in this paper?

My feeling is that the authors have something useful and interesting to say, but to make a persuasive case to which the wider scientific audience will pay attention and value requires a higher standard of presentation and rigour.

**We appreciate the constructive comments from the reviewer. To explain to the wider audience about the working group and its structure and how we approached the research and discussion, we added more information in the introduction p.3 “The multidisciplinary working group, AirPath…… in outdoor environments?” and “Participants were selected ….for the well being of society.”**

**In Research review and discussion, we added “methods” in it.**

**And a paragraph to explain the methods p. 4 - “At the first two meetings participants were given ….. connected to the five main areas arranged below.”**

**About the recent examples, the sentences were re-written p. 5. “Kuske reviewed the current and emerging technologies for the study of bacteria in the outdoor air in 2006 [5]. We searched the literatures from 2006 onwards….and paid close attention to the study of viruses.”**
